# Influenza Vaccination Coverage Among Health Care Personnel — United States, 2013–14 Influenza Season

**Published:** 2014-09-19

**Authors:** Carla L. Black, Xin Yue, Sarah W. Ball, Sara M.A. Donahue, David Izrael, Marie A. de Perio, A. Scott Laney, Megan C. Lindley, Samuel B. Graitcer, Peng-jun Lu, Walter W. Williams, Carolyn B. Bridges, Charles DiSogra, John Sokolowski, Deborah K. Walker, Stacie M. Greby

**Affiliations:** 1 Immunization Services Division, National Center for Immunization and Respiratory Diseases, CDC; 2 Abt Associates Inc., Cambridge, MA; 3 Division of Surveillance, Hazard Evaluations, and Field Studies, National Institute for Occupational Safety and Health, CDC; 4 Division of Respiratory Disease Studies, National Institute for Occupational Safety and Health, CDC; 5 Abt SRBI, New York, NY

The Advisory Committee on Immunization Practices recommends that all health care personnel (HCP) be vaccinated annually against influenza (1). Vaccination of HCP can reduce influenza-related morbidity and mortality among both HCP and their patients (1–4). To estimate influenza vaccination coverage among HCP during the 2013–14 season, CDC analyzed results of an opt-in Internet panel survey of 1,882 HCP conducted during April 1–16, 2014. Overall, 75.2% of participating HCP reported receiving an influenza vaccination during the 2013–14 season, similar to the 72.0% coverage among participating HCP reported in the 2012–13 season (5). Coverage was highest among HCP working in hospitals (89.6%) and lowest among HCP working in long-term care (LTC) settings (63.0%). By occupation, coverage was highest among physicians (92.2%), nurses (90.5%), nurse practitioners and physician assistants (89.6%), pharmacists (85.7%), and “other clinical personnel” (87.4%) compared with assistants and aides (57.7%) and nonclinical personnel (e.g., administrators, clerical support workers, janitors, and food service workers) (68.6%). HCP working in settings where vaccination was required had higher coverage (97.8%) compared with HCP working in settings where influenza vaccination was not required but promoted (72.4%) or settings where there was no requirement or promotion of vaccination (47.9%). Among HCP without an employer requirement for vaccination, coverage was higher for HCP working in settings where vaccination was offered on-site at no cost for 1 day (61.6%) or multiple days (80.4%) compared with HCP working in settings not offering free on-site vaccination (49.0%). Comprehensive vaccination strategies that include making vaccine available at no cost at the workplace along with active promotion of vaccination might be needed to increase vaccination coverage among HCP and minimize the risk for influenza to HCP and their patients.

The opt-in Internet panel survey was conducted for CDC by Abt Associates, Inc. (Cambridge, Massachusetts) during April 1–16, 2014, to provide estimates of influenza vaccination coverage among HCP during the 2013–14 influenza season. Two preexisting national opt-in Internet panels were used to recruit HCP for the survey. HCP were recruited through e-mails and messages on the panel websites and were eligible for the survey if they reported working in at least one of eight health care settings or reported any patient contact. Professional clinical HCP (physicians, nurse practitioners, physician assistants, nurses, dentists, pharmacists, allied health professionals, technicians, and technologists) were recruited from the current membership roster of Medscape, a medical website managed by WebMD Health Professional Network.* Medscape’s terms of service explicitly permit WebMD Professional Network to contact members about programming, including survey research; panelists receive an honorarium for completing surveys. HCP in other occupations (e.g., assistants, aides, administrators, clerical support workers, janitors, food service workers, and housekeepers) who met eligibility criterion were recruited for a health survey from general population Internet panels operated by or in partnership with Survey Sampling International (SSI) that provide panel members with online survey opportunities in exchange for nominal incentives.† Among the 2,054 HCP who entered the two panel survey sites and had eligible responses to the screening questions, 1,949 (94.9%) completed the survey.§ Sixty-six respondents with completed surveys who reported working in “other health care settings” were excluded because examination of other survey responses indicated that they were either unlikely to have contact with patients or that their work setting was not one of the health care settings of interest for this analysis. One respondent was excluded because of missing data for the question used to determine vaccination status, leaving a final analytic sample of 1,882 HCP.

Survey items included demographic characteristics, occupation type, work setting, self-reported influenza vaccination, and employer vaccination policies (vaccination requirements, vaccination availability at the workplace, and promotion of vaccination [including recognition, rewards, compensation, and free or subsidized vaccination]). Based on responses to the questionnaire, occupation type for HCP from both opt-in Internet panel sources were divided into seven groups for this analysis: physicians, nurse practitioners/physician assistants, nurses, pharmacists, assistants/aides, other clinical HCP, and nonclinical HCP. Work settings for HCP from both opt-in Internet panel sources were divided into four groups for this analysis: 1) hospitals, 2) ambulatory care/physician offices, 3) LTC settings, and 4) other clinical settings.¶ Respondents could specify working in more than one work setting. Sampling weights were calculated based on each occupation type by age, sex, race/ethnicity, work setting, and census region to represent the U.S. population of HCP. Vaccination coverage estimates from opt-in Internet panel surveys completed for the 2010–11, 2011–12, 2012–13, and 2013–14 seasons were compared to assess trends over time. Similar methodology was used for all four influenza seasons, although Internet panels used to recruit both clinical and nonclinical HCP in 2010–11 differed from those used in subsequent years (5–7). Because the study sample was based on HCP from opt-in Internet panels rather than probability samples, no statistical tests were performed.** Differences were noted when there was a difference of ≥5 percentage points between any estimates being compared.

What is already known on this topic?

The Advisory Committee on Immunization Practices recommends annual influenza vaccination for all health care personnel (HCP) to reduce influenza-related morbidity and mortality in health care settings. Estimates of overall HCP vaccination coverage were 66.9% for the 2011–12 season and 72.0% for the 2012–13 season.

What is added by this report?

Influenza vaccination coverage among HCP during the 2013–14 influenza season, assessed using an opt-in Internet panel survey, was 75.2%, similar to coverage for the 2012–13 season. Vaccination coverage was highest among physicians overall and HCP working in hospital settings; coverage was lowest among assistants/aides overall and HCP working in long-term care settings. Offering vaccination at the workplace at no cost was associated with higher vaccination coverage.

What are the implications for public health practice?

Comprehensive worksite intervention strategies that include vaccination promotion and convenient access to vaccination at no cost might increase vaccination coverage among HCP.

Overall, 75.2% of HCP reported receiving an influenza vaccination during the 2013–14 season, an increase of 11.7 percentage points compared with the 2010–11 season estimate, but similar to the 72.0% coverage estimate reported in 2012–13 (Figure, Table 1). With the exception of LTC settings, coverage for the 2013–14 season was higher in all work settings compared with the 2010–11 and 2011–12 seasons (Figure). However, overall, only HCP working in hospital settings had an increase in coverage during the 2013–14 season compared with the 2012–13 season (Figure, Table 1). By occupation type, coverage during the 2013–14 season was 92.2% among physicians, 89.6% among nurse practitioners/physician assistants, 90.5% among nurses, 85.7% among pharmacists, 57.7% among assistants/aides, 87.4% among other clinical personnel, and 68.6% among nonclinical personnel (Table 1). Only nurses and other clinical personnel had increased coverage compared with the 2012–13 season (90.5% versus 84.8% and 87.4% versus 81.9%, respectively).

During the 2013–14 season, influenza vaccination coverage was higher for HCP working in settings where vaccination was required (97.8%) compared with HCP working in settings where vaccination was not required but promoted (72.4%) or settings where there was no requirement or promotion (47.9%) (Table 2). Influenza vaccination coverage was above 96% in all work settings where vaccination was required, including LTC settings (Table 2). Thirty-six percent of HCP were required by their employer to be vaccinated, an increase from 13% during the 2010–11 season. HCP working in hospitals were more likely to be required to be vaccinated (58.2%) than those working in other settings (range = 20.1%–33.6%), whereas HCP working in LTC settings were least likely to be required to be vaccinated (20.1%). HCP working in LTC settings were most likely to report that their employer neither required nor promoted vaccination (42.6%) compared with HCP working in other health care settings. In contrast, only 7.0% of HCP working in hospitals reported that their employer neither required nor promoted vaccination (Table 2).

The majority of vaccinated HCP (77.3%) reported receiving the vaccination at work. Among HCP without an employer requirement for vaccination, vaccination coverage among HCP working in facilities that made vaccination available on-site at no cost for more than 1 day was 80.4%, compared with 61.6% in facilities that made vaccination available at no cost for 1 day only and 49.0% in facilities that did not provide influenza vaccination on-site or offered on-site vaccination but not at no cost (Table 2). On-site vaccination for more than 1 day at no cost was more likely to be available to HCP working in hospitals (75.1%) than to HCP working in ambulatory care settings (43.0%), other clinical settings (31.1%), and LTC settings (14.6%).

Among vaccinated HCP, the most common reasons given for vaccination were “To protect myself from flu” (43.5%), “My employer requires me to be vaccinated for flu” (25.5%), and “To protect patients from getting flu” (8.5%). Among unvaccinated HCP, the most common reasons given for not being vaccinated†† were “I might get sick from the vaccine” (20.1%), “I don’t think that flu vaccines work” (16.3%), and “I don’t need it” (16.0%).

## Discussion

The overall HCP influenza vaccination coverage estimate for the 2013–14 season was 75.2%, similar to the estimate of 72.0% from the previous influenza season, but higher than the estimates of 63.5% and 66.9% observed for the 2010–11 and 2011–12 seasons, respectively (5–7). As in the 2012–13 season, coverage during the 2013–14 season was >90% for two groups of HCP: physicians, regardless of the settings in which they worked, and HCP with an employer requirement to be vaccinated, regardless of work setting.

The results of this survey showed that higher vaccination coverage among HCP was associated with employer vaccination requirements, vaccination promotion, and access to vaccination at the workplace at no cost for more than 1 day. Vaccination at the worksite, the most common place of vaccination reported by HCP in this survey, has been associated with higher seasonal vaccination coverage among HCP (8). This study found that coverage of 80.4% was achieved in the absence of a vaccination requirement among HCP working in facilities where free on-site vaccination was available for more than 1 day. However, 49.3% of HCP without a requirement to be vaccinated worked in locations that either did not offer vaccination on-site, or if offered, did not make vaccination available at no cost. These results indicate that a comprehensive strategy that includes promotion of vaccination along with easy access to vaccination at no cost on multiple days might increase HCP vaccination coverage.

Consistent with the previous two seasons, coverage among HCP working in LTC settings was the lowest among the work settings examined. This might be attributable to several factors. The majority of surveyed HCP working in LTC settings were assistants or aides, the occupational group with the lowest coverage in this analysis. HCP working in LTC settings also were most likely to report that their employer neither required nor promoted vaccination and least likely to report that their employer made vaccination available at no cost for multiple days. Influenza vaccination of HCP in LTC settings is important given that influenza vaccine effectiveness is generally lowest in the elderly, making vaccination of close contacts even more critical (3). In addition, multiple studies have demonstrated that vaccination of HCP in LTC settings confers a health benefit to patients, including reduced risk for mortality (2–4).

The findings in this report are subject to at least six limitations. First, the findings might differ from those based on the National Health Interview Survey (NHIS), a probability-based survey that might be more representative of the general HCP personnel population. Influenza vaccination coverage among HCP from the opt-in Internet panel survey differed from the population-based sample in the NHIS in the 2009–10 through 2011–12 seasons (e.g., 66.9% influenza vaccination coverage among HCP in the opt-in Internet panel survey versus 62.4% in the NHIS for the 2011–12 season) (9). Additional comparisons with NHIS and other available data sources over multiple seasons are needed to determine whether the more timely opt-in Internet panel survey estimates, despite sampling differences, provide valid assessments of trends. Second, the sample was not randomly selected from HCP in the United States. The opt-in Internet panel survey used a nonprobability sample of volunteer HCP members of the Medscape and SSI Internet panels and did not include HCP without Internet access. Despite poststratification weighting, the results based on this nonprobability sample might not be representative of the HCP population in the United States. Noncoverage and nonresponse bias might remain even after weighting adjustments. Third, all results were based on self-report and were not verified by employment or medical records. Self-report of vaccination might be subject to recall bias. Fourth, the wording of the questions used to ascertain vaccination promotion in the 2012–13 and 2013–14 surveys differed from previous surveys; therefore, the vaccination promotion trend might not be comparable across survey years. Fifth, the 2011–12 through 2013–14 opt-in Internet panel survey might not be directly comparable with the 2010–11 opt-in Internet panel survey because different methods of recruitment were used for the 2010–11 season. Whereas the same two opt-in Internet panels were used for the 2011–12 through 2013–14 surveys, the majority of HCP in the 2010–11 survey were recruited from a population-based Internet research panel that was supplemented with respondents from opt-in medical specialty and general population Internet panels that differed from those used in subsequent years (5–7). Sixth, the definitions of some occupational groups differ across survey years; dentists were included with physicians in the 2010–11 survey, and pharmacists were included with “other clinical personnel” prior to the 2012–13 survey. Finally, the definitions of HCP, occupation type, and work setting used in this opt-in Internet panel survey vary from definitions used in other surveys of vaccination coverage; therefore, results might not be comparable.

The *Guide to Community Preventive Services*§§ provides guidance on effective evidence-based interventions to increase the use of influenza vaccination. Higher vaccination coverage and increased use of vaccination requirements and promotion in hospitals compared with other settings might be partly attributable to the Centers for Medicare & Medicaid Services requirement to report HCP influenza vaccination levels as part of the Hospital Inpatient Quality Reporting Program (10). The results of this report indicate that higher HCP vaccination coverage was associated with employer requirements for vaccination. In the absence of vaccination requirements, expanding the number of health care facilities offering vaccination on-site, over multiple days, and at no cost might help sustain and improve influenza vaccination coverage among HCP.

## Figures and Tables

**FIGURE:**
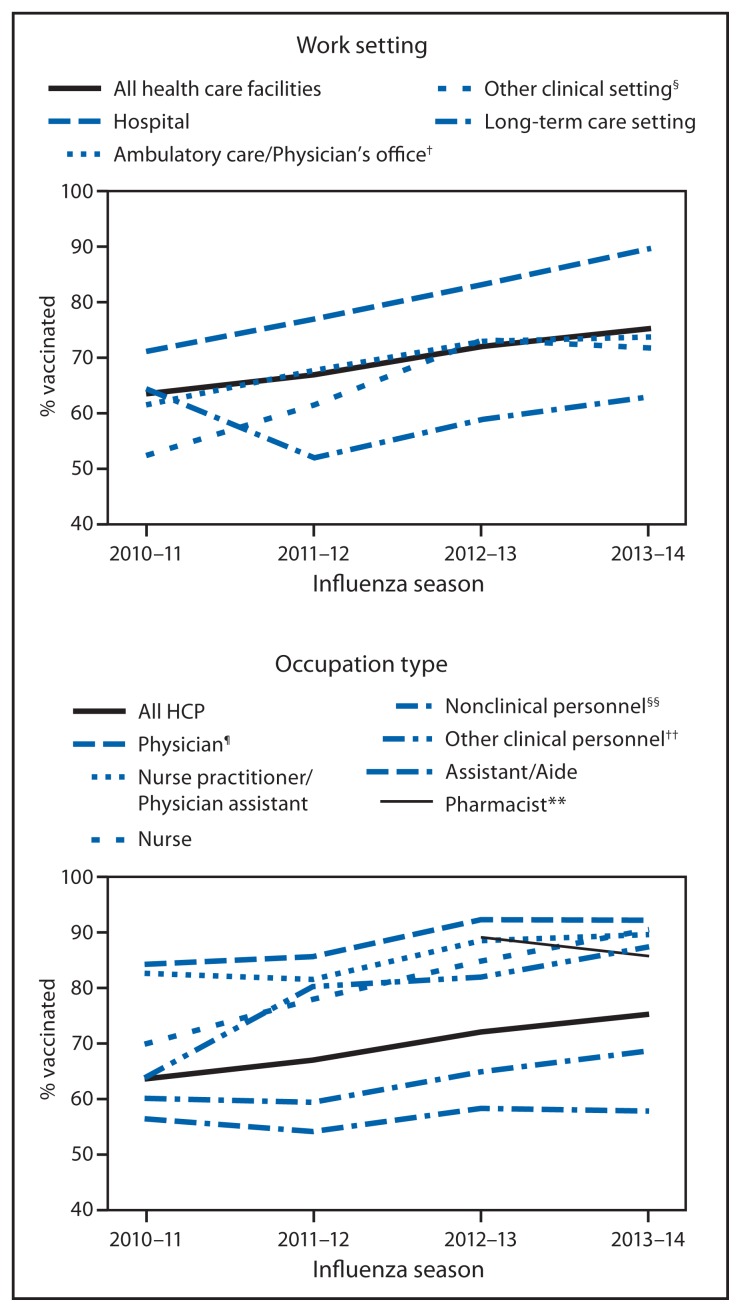
Percentage of health-care personnel (HCP)* who received influenza vaccination, by work setting and occupation type — Internet panel survey, United States, 2010–11 through 2013–14 influenza seasons * Persons who work in a place where clinical care or related services were provided to patients, or whose work involves face-to-face contact with patients or who were ever in the same room as patients. ^†^ Ambulatory care (physician’s office, medical clinic, and other ambulatory care setting). ^§^ Dentist’s office or dental clinic, pharmacy, laboratory, public health setting, health care education setting, emergency medical services setting, or other setting where clinical care or related services was provided to patients. ^¶^ Included dentists for 2010–11 season. ** Individual data on pharmacists not collected before the 2012–13 season. ^††^ Allied health professionals, technicians, and technologists. Includes pharmacists for the 2010–11 and 2011–12 seasons. ^§§^ Administrative support staff or managers and nonclinical support staff (e.g., food service workers, housekeeping staff, maintenance staff, janitors, and laundry workers).

**TABLE 1:** Percentage of health care personnel (HCP)* who received influenza vaccination, by work setting and occupation — Internet panel survey, United States, 2012–13 and 2013–14 influenza seasons

Work setting†/Occupation	2012–13			2013–14			Percentage-point difference 2012–13 to 2013–14
	
	No. in sample	Weighted %§	Weighted % vaccinated	No. in sample	Weighted %§	Weighted % vaccinated	
**Overall**	**1,944**	**100.0**	**72.0**	**1,882**	**100.0**	**75.2**	**3.2**
Hospital	961	38.1	83.1	880	40.8	89.6	6.5
Physician	209	6.3	93.2	185	5.7	93.1	−0.1
NP/PA	50	1.3	88.0	42	0.8	97.6	9.6
Nurse	121	28.2	86.5	125	27.5	94.7	8.2
Pharmacist	44	0.6	97.7	—¶	—¶	—¶	—¶
Assistant/Aide	—¶	—¶	—¶	—¶	—¶	—¶	—¶
Other clinical personnel**	328	25.1	88.1	271	28.3	92.3	4.2
Nonclinical personnel††	177	27.2	79.5	198	29.2	84.4	4.9
**Ambulatory care/Physician office** §§	636	32.9	72.9	649	28.0	73.7	0.8
Physician	221	9.4	91.6	250	11.4	91.6	0.0
NP/PA	94	2.9	92.6	98	2.6	89.8	−2.8
Nurse	48	18.0	79.9	56	19.4	82.2	2.3
Pharmacist	—¶	—¶	—¶	—¶	—¶	—¶	—¶
Assistant/Aide	—¶	—¶	—¶	—¶	—¶	—¶	—¶
Other clinical personnel**	163	22.2	80.3	125	18.2	78.9	−1.4
Nonclinical personnel††	79	36.1	58.6	90	40.7	62.7	4.1
**Long-term care setting**	427	22.8	58.9	364	30.5	63.0	4.1
Physician	—¶	—¶	—¶	—¶	—¶	—¶	—¶
NP/PA	—¶	—¶	—¶	—¶	—¶	—¶	—¶
Nurse	32	7.5	85.4	—¶	—¶	—¶	—¶
Pharmacist	—¶	—¶	—¶	—¶	—¶	—¶	—¶
Assistant/Aide	143	70.7	54.7	121	60.9	54.4	−0.3
Other clinical personnel**	52	4.1	65.7	47	6.2	89.4	23.7
Nonclinical personnel††	165	16.1	60.8	139	22.6	66.4	5.6
**Other clinical setting** ¶¶	237	15.1	73.2	327	11.9	71.7	−1.5
Physician	—¶	—¶	—¶	—¶	—¶	—¶	—¶
NP/PA	—¶	—¶	—¶	—¶	—¶	—¶	—¶
Nurse	—¶	—¶	—¶	—¶	—¶	—¶	—¶
Pharmacist	61	2.2	88.5	64	9.4	87.6	−0.9
Assistant/Aide	—¶	—¶	—¶	—¶	—¶	—¶	—¶
Other clinical personnel**	74	25.5	75.0	167	27.0	82.0	7.0
Nonclinical personnel††	46	35.1	56.7	46	34.3	52.1	−4.6
**Overall occupation**							
Physician	322	4.3	92.3	326	4.1	92.2	−0.1
NP/PA	131	1.3	88.5	125	0.9	89.6	1.1
Nurse	202	19.4	84.8	203	18.7	90.5	5.7
Pharmacist	92	0.5	89.1	75	1.3	85.7	−3.4
Assistant/Aide	178	24.7	58.2	152	23.6	57.7	−0.5
Other clinical personnel**	544	19.2	81.9	533	19.3	87.4	5.5
Nonclinical personnel††	449	30.2	64.8	445	31.9	68.6	3.8

**Abbreviation:** NP/PA = nurse practitioner/physician assistant.

* Persons who work in a place where clinical care or related services were provided to patients, or whose work involves face-to-face contact with patients or who were ever in the same room as patients.

† Respondents could specify working in more than one setting.

§ Weights were calculated based on each occupation type by age, sex, race/ethnicity, work setting, and census region to represent the U.S. population of HCP. Work setting and overall occupation are presented as weighted estimates of the total sample. Where the groups are stratified by work setting, the weighted estimates are presented for each subgroup within the group.

¶ Estimate suppressed because sample size was <30.

** Allied health professionals, technicians, and technologists.

†† Administrative support staff or managers and nonclinical support staff (e.g., food service workers, housekeeping staff, maintenance staff, janitors, and laundry workers).

§§ Physician’s offices, medical clinics, and other ambulatory care settings.

¶¶ Dentist office or dental clinic, pharmacy, laboratory, public health setting, health care education setting, emergency medical services setting, or other settings where clinical care or related services was provided to patients.

**TABLE 2:** Percentage of health care personnel (HCP)* who received influenza vaccination, by work setting and vaccination requirements/availability status — Internet panel survey, United States, 2010–11 through 2013–14 influenza seasons

Vaccination status/Work setting	2010–11			2011–12			2012–13			2013–14		
			
	No. in sample	Weighted %†	Weighted % vaccinated	No. in sample	Weighted %†	Weighted % vaccinated	No. in sample	Weighted %†	Weighted % vaccinated	No. in sample	Weighted %†	Weighted % vaccinated
**Influenza vaccination requirement and vaccine promotion (2012–13 season definition)**												
**Required**	230	13.0	98.1	496	21.1	93.7	549	22.4	96.5	738	35.5	97.8
Hospital	121	21.9	98.1	362	32.0	95.2	388	37.2	95.1	520	58.2	97.7
Ambulatory care/Physician office§	76	11.0	96.2	153	21.8	95.5	191	21.0	99.8	252	33.6	96.4
Long-term care	—¶	—¶	—¶	45	10.4	86.1	61	13.0	95.8	88	20.1	98.4
Other clinical setting**	—¶	—¶	—¶	—¶	—¶	—¶	38	10.7	100.0	88	29.3	99.5
**No requirement but promotion** ††	320	17.2	64.8	390	16.1	75.4	901	43.1	76.9	764	38.6	72.4
Hospital	141	22.3	62.0	255	21.6	75.4	456	49.7	78.1	307	34.8	79.8
Ambulatory care/Physician office§	88	13.9	60.2	106	15.3	70.0	273	41.2	80.1	247	40.2	73.6
Long-term care	31	18.1	71.9	62	14.0	77.7	183	35.0	67.0	149	37.3	61.5
Other clinical setting**	60	11.0	71.8	30	8.9	94.9	134	49.9	85.7	164	43.7	71.9
**No requirement or promotion**	1,373	69.9	56.7	1,450	62.8	55.2	487	34.5	50.4	378	25.6	47.9
Hospital	352	55.8	64.2	566	46.4	65.0	115	13.1	67.7	53	7.0	70.3
Ambulatory care/Physician office§	490	75.1	56.5	486	62.9	57.0	170	37.9	50.4	150	26.2	44.5
Long-term care	173	73.2	58.2	343	75.6	41.4	179	52.0	45.0	127	42.6	47.7
Other clinical setting**	358	86.2	48.4	225	84.0	56.5	65	39.5	50.2	75	26.9	41.0
**Influenza vaccination available at no cost** §§												
**For more than 1 day** ¶¶	1,095	60.1	69.6	971	44.2	71.6	658	36.7	80.5	542	38.9	80.4
Hospital	436	86.5	69.0	598	69.7	72.3	382	59.4	81.9	261	75.1	82.0
Ambulatory care/Physician office§	385	62.4	70.4	314	41.8	70.0	189	35.2	82.3	183	43.0	80.7
Long-term care	121	55.4	71.0	124	22.8	61.2	115	19.8	74.8	63	14.6	71.6
Other clinical setting**	153	27.9	69.3	88	30.1	87.1	85	32.0	84.3	107	31.1	85.0
**1 day only** ¶¶	64	4.8	46.0	245	15.1	59.9	227	12.7	67.6	169	11.8	61.6
Hospital	—¶	—¶	—¶	104	14.9	60.1	89	15.8	66.3	43	10.0	55.6
Ambulatory care/Physician office§	—¶	—¶	—¶	82	15.8	52.8	88	11.8	80.1	76	17.0	69.3
Long-term care	—¶	—¶	—¶	48	14.9	53.2	58	12.9	49.7	43	12.5	54.1
Other clinical setting**	—¶	—¶	—¶	40	13.6	89.7	—¶	—¶	—¶	31	9.2	72.9
**Not available** ***	529	35.0	40.4	622	40.7	45.0	510	50.6	53.1	433	49.3	49.0
Hospital	41	9.4	33.9	120	15.5	56.9	102	24.9	67.9	56	15.0	74.5
Ambulatory care/Physician office§	175	32.9	28.7	197	42.4	51.3	168	53.0	51.6	138	40.0	39.1
Long-term care	67	37.0	51.4	231	62.3	39.1	193	67.3	47.8	170	72.9	50.6
Other clinical setting**	246	67.8	42.2	125	56.3	39.4	89	59.8	60.7	101	59.7	45.1

* Persons who work in a place where clinical care or related services were provided to patients, or whose work involves face-to-face contact with patients or who were ever in the same room as patients.

† Weights were calculated based on each occupation type by age, sex, race/ethnicity, work setting, and census region to represent the U.S. population of HCP. Requirement status and vaccine availability are presented as weighted estimates of the total sample. Where the groups are stratified by work setting, the estimates are presented as weighted estimates of the subsample of each work setting subgroup.

§ Physician’s offices, medical clinics, and other ambulatory care settings.

¶ Estimate suppressed because sample size was <30.

** Dentist office or dental clinic, pharmacy, laboratory, public health setting, health care education setting, emergency medical services setting, or other setting where clinical care or related services was provided to patients.

†† Influenza vaccination was promoted among employees through public identification of vaccinated persons, financial incentives or rewards to groups of employees, competition among units or care areas, free or subsidized cost of vaccination, reminders, publicizing of the number or percentage of employees receiving vaccination, and special events.

§§ Restricted to respondents without a requirement for vaccination. In the 2013–14 season, 87.9% of HCP with a requirement for vaccination had vaccination available onsite at no cost for at least 1 day. Vaccination coverage among HCP with a vaccination requirement was >96%, regardless of workplace availability of vaccination.

¶¶ Question only asked of those reporting influenza vaccinations offered on-site during this influenza season.

*** Influenza vaccination not offered on-site during the influenza season or offered on-site but not available at no cost to employees.

## Footnotes

* Additional information available at http://www.medscape.com.

† Additional information available at http://www.surveysampling.com.

§ A survey response rate requires specification of the denominator at each stage of sampling. During recruitment of an online opt-in survey sample, such as the Internet panels described in this report, these numbers are not available; therefore, a response rate cannot be calculated. Instead, the survey completion rate is provided.

¶ Ambulatory care/physician’s office included physicians’ offices, medical clinics, and other nonhospital outpatient or ambulatory care settings. LTC settings included nursing homes, home health agencies, home health care settings, assisted living facilities, or other LTC settings. Other clinical setting included dental offices or clinics, pharmacies, laboratories, public health settings, health care education settings, emergency medical services settings, or other settings where clinical care or related services were provided to patients.

** Additional information available at http://www.aapor.org/opt_in_surveys_and_margin_of_error1.htm.

†† Among respondents, 28.4% initially selected “I just don’t want the vaccine” as their main reason for not being vaccinated. When these persons were asked in a follow-up question to identify a more specific reason for not wanting the vaccine, 28.0% replied “I don’t think the ingredients in the vaccine are good for you,” 27.8% said “I don’t need it,” 23.2% said “I might get sick from the vaccine,” and 20.9% said “I don’t think that flu vaccines work.”

§§ Additional information available at http://www.thecommunityguide.org/vaccines/index.html.
